# Cohort profile: Evaluation of the Methods and Management of Acute Coronary Events (EMMACE) longitudinal cohort

**DOI:** 10.1093/ehjqcco/qcad040

**Published:** 2023-07-14

**Authors:** Theresa Munyombwe, Tatendashe B Dondo, Marlous Hall, Ramesh Nadarajah, Ben Hurdus, Suleman Aktaa, Mohammad Haris, Adam Keeley, Robert West, Alistair Hall, Anzhela Soloveva, Paul Norman, Chris P Gale

**Affiliations:** Leeds Institute of Cardiovascular and Metabolic Medicine, University of Leeds, 6 Clarendon Way, LS2 9JT Leeds, UK; Leeds Institute for Data Analytics, University of Leeds, LS2 9JT Leeds, UK; Leeds Institute of Cardiovascular and Metabolic Medicine, University of Leeds, 6 Clarendon Way, LS2 9JT Leeds, UK; Leeds Institute for Data Analytics, University of Leeds, LS2 9JT Leeds, UK; Leeds Institute of Cardiovascular and Metabolic Medicine, University of Leeds, 6 Clarendon Way, LS2 9JT Leeds, UK; Leeds Institute for Data Analytics, University of Leeds, LS2 9JT Leeds, UK; Leeds Institute of Cardiovascular and Metabolic Medicine, University of Leeds, 6 Clarendon Way, LS2 9JT Leeds, UK; Leeds Institute for Data Analytics, University of Leeds, LS2 9JT Leeds, UK; Department of Cardiology, Leeds Teaching Hospitals NHS Trust, LS1 3EX Leeds, UK; Leeds Institute for Data Analytics, University of Leeds, LS2 9JT Leeds, UK; Leeds Institute for Data Analytics, University of Leeds, LS2 9JT Leeds, UK; Department of Cardiology, Leeds Teaching Hospitals NHS Trust, LS1 3EX Leeds, UK; Leeds Institute for Data Analytics, University of Leeds, LS2 9JT Leeds, UK; Department of Cardiology, Leeds Teaching Hospitals NHS Trust, LS1 3EX Leeds, UK; Leeds Institute for Data Analytics, University of Leeds, LS2 9JT Leeds, UK; Leeds Institute of Health Sciences, University of Leeds, LS2 9JT Leeds, UK; Leeds Institute of Cardiovascular and Metabolic Medicine, University of Leeds, 6 Clarendon Way, LS2 9JT Leeds, UK; Department of Cardiology, Leeds Teaching Hospitals NHS Trust, LS1 3EX Leeds, UK; Department of Cardiology, Almazov National Medical Research Centre, 2 Akkuratova street, Saint Petersburg 197341, Russian; School of Geography, University of Leeds, LS2 9JT Leeds, UK; Leeds Institute of Cardiovascular and Metabolic Medicine, University of Leeds, 6 Clarendon Way, LS2 9JT Leeds, UK; Leeds Institute for Data Analytics, University of Leeds, LS2 9JT Leeds, UK; Department of Cardiology, Leeds Teaching Hospitals NHS Trust, LS1 3EX Leeds, UK

**Keywords:** Myocardial infarction, Outcomes, Hospitalizations, Medications, Health-related quality of life, Cohort, Electronic health records, Longitudinal data

## Abstract

**Aims:**

The Evaluation of the Methods and Management of Acute Coronary Events (EMMACE) longitudinal cohort study aims to investigate health trajectories of individuals following hospitalization for myocardial infarction (MI).

**Methods and results:**

EMMACE is a linked multicentre prospective cohort study of 14 899 patients with MI admitted to 77 hospitals in England who participated in the EMMACE-3 and -4 studies between 1st November 2011 and 24th June 2015. Long-term follow-up of the EMMACE cohorts was conducted through the EMMACE-XL (27th September 2020 to 31st March 2022) and EMMACE-XXL (1st July 2021 to 1st July 2023) studies. EMMACE collected individual participant data for health-related quality of life (HRQoL) measured by three-level EuroQol five-dimension and visual analogy scale at admission, 1 month, 6 months, 12 months, and 10 years follow-up, as well as medications, medication adherence, beliefs about medicines, Satisfaction with Information about Medicines Scale, and illness perceptions. Participant data were deterministically linked to the Myocardial Infarction National Audit Project (MINAP) for information on baseline treatments and comorbidities, Hospital Episode Statistics Admitted Patient Care (for cause-specific hospitalization data), and the Office for National Statistics (for mortality data) up to 2020.

**Conclusion:**

EMMACE is a nationwide prospective cohort that will provide unique insights into fatal and non-fatal outcomes, medication adherence, and HRQoL following MI.

Trial registration: ClinicalTrials.gov NCT01808027 and NCT01819103

**Key learning points**

What is already known

- Health-related quality of life (HRQoL) is a key outcome in cardiovascular diseases. Researchers have focused mostly on objective measures of health, such as mortality and morbidity, but HRQoL may impact negatively on patient outcomes.

What this study adds

- This is the largest nationwide patient-level cohort to date that provides a holistic view on health states of myocardial infarction (MI) survivors capturing HRQoL and clinical outcome trends up to 10 years following hospitalization with MI. The use of a longitudinal study design allows the capture of temporal trends in changes of HRQoL over time and associations with patient outcomes.
- This cohort profile data can be used to determine the associations of HRQoL with fatal and non-fatal outcomes following MI identifying precisely in whom worse (or better) outcomes may occur to permit the design and testing of novel interventions to reduce premature death from MI and its complications.

## Introduction

Cardiovascular disease is the leading cause of death globally,^1^ contributing to a third of all deaths and reduced quality of life.^2^ Despite a substantial decline in mortality rates from cardiovascular disease,^3–5^ myocardial infarction (MI) remains unnecessarily common and, in addition to its death toll and economic burden, is associated with a legacy of recurrent cardiovascular events, including heart failure, cerebrovascular disease, and MI.^2,6^

Information about the health outcomes of people with MI is required to determine individual health needs, enable earlier detection and treatment of new onset disease, and inform health service planning. However, there is a limited literature about health outcomes of people with MI that is nationally representative, includes patient survey data as well as information systematically collected through electronic health records (EHRs), and extends many years from the index admission MI. In some geographies, fatal and non-fatal cardiovascular events after MI have now reached a plateau, yet remain elevated beyond the first year.^7–9^ This suggests a need for prolonged surveillance of individuals with MI and a refreshed perspective on the outcomes from MI and their management. This paper provides a cohort profile of the Evaluation of the Methods and Management of Acute Coronary Events (EMMACE) longitudinal cohort study containing long-term survey and EHRs follow-up data for individuals with MI across 77 hospitals in England. The EMMACE-3 and -4 studies^10^ and the EMMACE-XL and EMMACE-XXL follow-up studies were designed to collect individual participant data about a wide range of clinical outcomes following admission with MI.

### Aim of the EMMACE longitudinal cohort

The aim of the EMMACE longitudinal cohort is to collect and study longitudinal health outcomes in individuals admitted with MI, providing unique insights into patient health trajectories after MI, including healthcare utilization, health-related (HRQoL), and how they may change over time.

### Quality-of-care interventions

The EMMACE longitudinal cohort builds on the successes of EMMACE-1 and -2, which were used for translational and cardiovascular outcomes research.^11–21^ Individual patient data were collected across healthcare utilization, HRQoL, and how they changed over time. As the registry aims to capture real-world outcomes for patients who had experienced an MI, management and/or treatment management strategies were not pre-specified by the study protocol.

### Study setting

**Figure 1:**
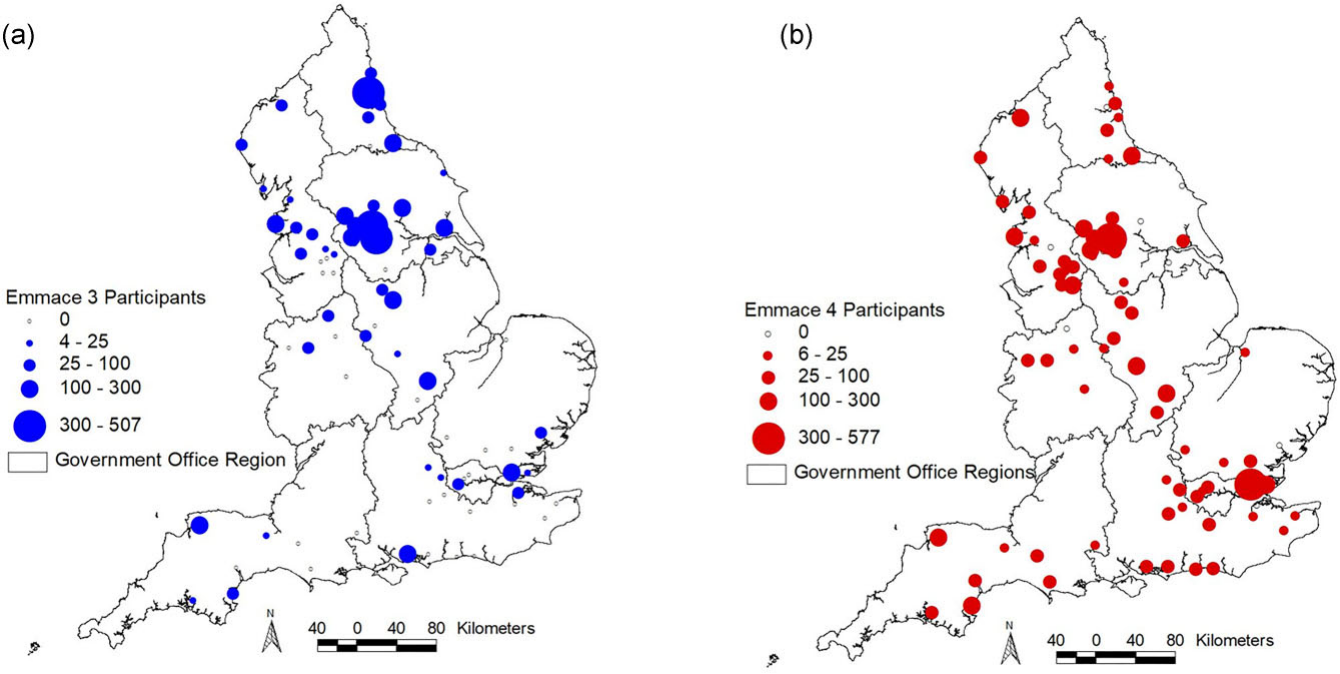
Regional maps of English National Health Service hospitals and patients participating in the Evaluation of the Methods and Management of Acute Events [EMMACE]-3 and 4.

A total of 77 National Health Service (NHS) hospitals in England (*Figure 1*) participated in the study, which comprises data from two sequential recruiting cohorts—EMMACE-3 and EMMACE-4.

### Populations and consent

EMMACE-3 (*n* = 5556) participants were recruited between 1st November 2011 and 17th September 2013 and EMMACE-4 (*n* = 9343) participants were recruited between 1st October 2013 and 24th June 2015. Long-term follow-up of the EMMACE cohorts was conducted through the EMMACE-XL (27th September 2020 to 31st March 2022) and EMMACE-XXL (1st July 2021 to 1st July 2023).

Participants consented to enter the EMMACE-3 and -4 studies and for their data to be linked to other datasets and shared. The EMMACE-3 and -4 studies were given ethics approval by the Leeds (West) Research Ethics Committee (REC reference: 10/H131374, 13/YH/0277, and 12/WM/0431). The EMMACE-XL and EMMACE-XXL studies were granted ethics approval by the London Bridge Research Ethics Committee (EMMACE-XL, 20/PR/0104) and by London-Hampstead Research ethics committee (EMMACE-XXL, 21/PR/0810). Permissions for the linked Hospital Episode Statistics Admitted Patient Care (HES APC) data and ONS mortality data were obtained through NHS Digital (DARS-NIC-332338-X1N2G-v0.9).

### Start points

The EMMACE studies included patients aged 18 years or older who had been admitted with an acute coronary syndrome at one of the participating hospitals. Patients at a terminal stage of any illness, and those for whom follow-up would be inappropriate or impractical (e.g. patients requiring emergency treatment), were excluded from the study.

### Baseline and follow-up data

Baseline data included patient demographics and socioeconomic status, patient-reported data at the time of admission with MI concerning HRQoL (the three-level EuroQol five-dimension [EQ-5D-3L],^22^ EQ visual analogue scale [EQ-VAS]), treatments, medication beliefs questionnaire,^23^ Single Question Medicine Adherence, Satisfaction with Information about Medicines Scale [SIMS],^24^ Care Quality Commission Pickering Inpatient questionnaire,^25^ and Brief Illness Perception.^26^ Baseline co-morbidities and quality-of-care data were obtained from the Myocardial Infarction National Audit Project (MINAP) registry.^27^

**Figure 2:**
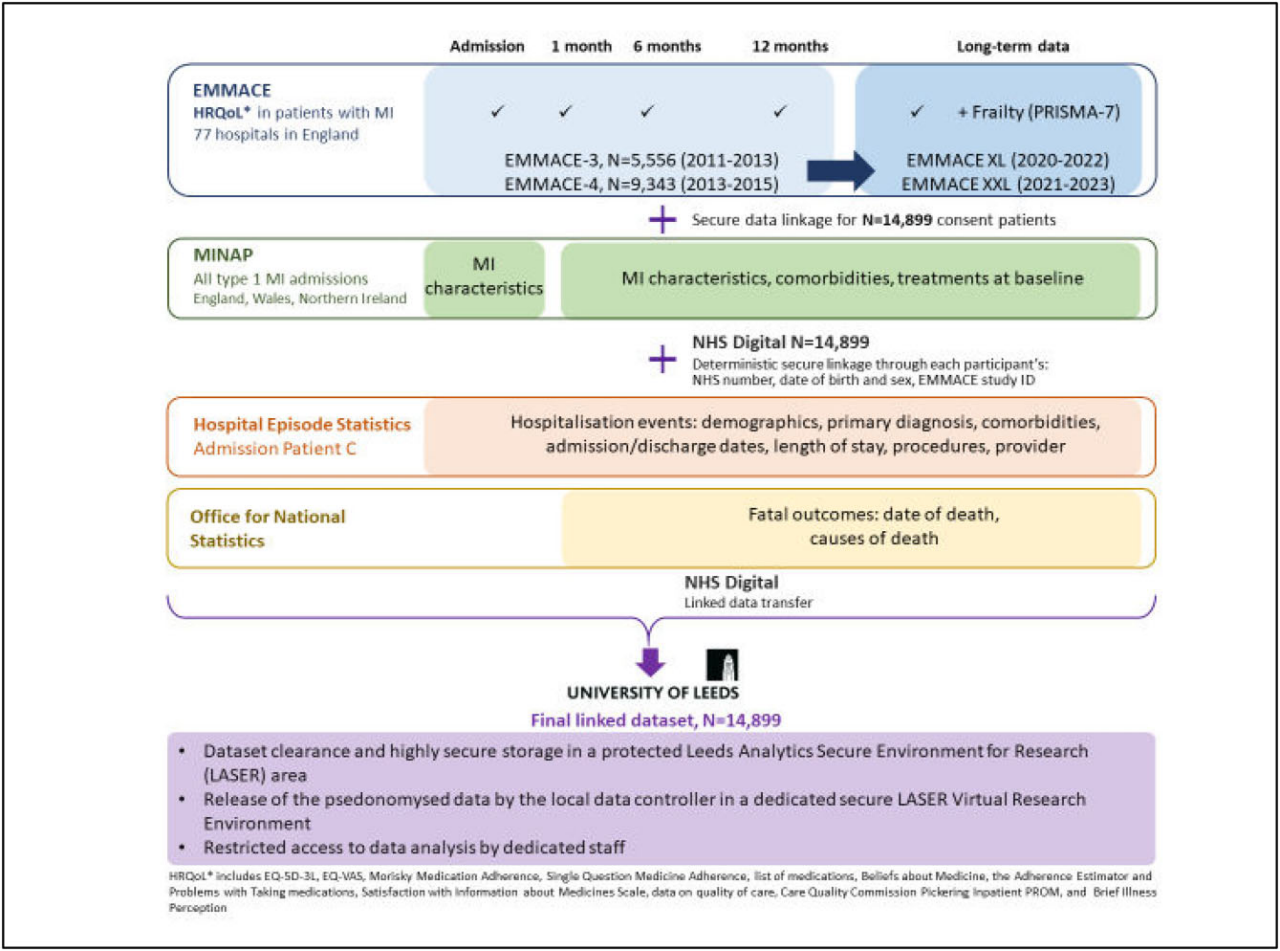
The EMMACE longitudinal cohort data flow and linkages.

Follow-up of HRQoL measured by EQ-5D-3L and EQ-VAS was recorded at 1 month, 6 months, and 12 months. At 10 years, through the EMMACE-XL study (2020–21), the EMMACE cohort participants were contacted and consented to complete a further quality-of-life questionnaire (EQ-5D-3L, EQ-VAS), supply their current medication data, and answer a question relating to their perceived adherence to their current medication as well as to answer lifestyle questions about their smoking habits. An additional follow-up of the EMMACE cohort was through the EMMACE- XXL(2021–22) study that collected further quality-of-life data (EQ-5D-3L, EQ-VAS), information about frailty (using the Program of Research on Integration of Services for the Maintenance of Autonomy [PRISMA-7] frailty questionnaire),^28^ and the Single Question Medicine Adherence tool. Details of the EMMACE data flow are shown in *Figure 2*, and variables in the EMMACE dataset are shown in Supplementary material online, *Table S1*.

### Data quality and linkages

Consented EMMACE-3 and -4 participants (*n* = 14 899) were linked to the MINAP registry^27^ with a further deterministic linkage with HES-APC^29^ data and the Office for National Statistics for the years 2010/11 to 2020/21 by NHS Digital acting as the trusted third party (DARS-NIC-332338-X1N2G-v0.9). Each participant's NHS number, date of birth, and sex along with their EMMACE study identification number was securely transmitted to NHS Digital whereby returned data included (for patients with prior and/or subsequent hospitalizations) corresponding HES records, cause of death, and date of death for patients who have died. NHS Digital publishes how their databases are regularly checked to ensure accuracy of the recorded data, and their methods of data cleaning and quality assurance.^30^ Details of the EMMACE data flow are shown in *Figure 2*.

### Data capture and storage

The data are considered highly confidential as they contain identifiable patient-level data linked with EHRs, clinical management data and their storage and sharing abide by the University of Leeds data protection and sharing policies. Data are stored in the Leeds Analytics Secure Environment for Research (LASER) within the University of Leeds. LASER is a Leeds Institute for Data Analytics purpose-built cloud-based platform for hosting sensitive data compliant with ISO 27001 standards and the NHS Data security and protection toolkit. Pseudonymized data are accessible for analysis with approval from the local data controller (CPG) in a dedicated secure LASER Virtual Research Environment. All individuals with access to these data are required to undergo the University of Leeds information security training and to sign an information security policy before accessing the data. The University of Leeds Information Security Policy is implemented and drawn up in line with ISO 27 001.

### Access to data

Access to the EMMACE longitudinal cohort data may be requested by contacting the Chief Investigator (CPG).

## Conclusion

The EMMACE longitudinal cohort is a nationwide individual participant-level database that provides a holistic view of the states of health of people with MI, capturing HRQoL, medications, all hospitalized events, and all deaths during 10 years of follow-up. The real-world origin and robustness of the data sources provide a strong basis for the generalizability of the study results, which can be translated into novel post-MI healthcare system goals. Phenotyping MI survivors based on longitudinal changes in HRQoL and subsequent outcomes may allow healthcare providers to identify high-risk group of patients who may benefit from timely targeted interventions to achieve sustained improvements in health status.

## Supplementary Material

qcad040_Supplemental_File

## Data Availability

The data underlying this article will be shared on reasonable request to the Chief Investigator (C.P.G.).
