# Corrigendum to: Cohort profile: Evaluation of the Methods and Management of Acute Coronary Events (EMMACE) longitudinal cohort

**DOI:** 10.1093/ehjqcco/qcad066

**Published:** 2023-11-30

This is a corrigendum to: Theresa Munyombwe, Tatendashe B. Dondo, Marlous Hall, Ramesh Nadarajah, Ben Hurdus, Suleman Aktaa, Mohammad Haris, Adam Keeley, Robert West, Alistair Hall, Anzhela Soloveva, Paul Norman, Chris P. Gale, Cohort profile: Evaluation of the Methods and Management of Acute Coronary Events (EMMACE) Longitudinal Cohort, *European Heart Journal—Quality of Care and Clinical Outcomes*, Volume 9, Issue 5, August 2023, Pages 442–446, https://doi.org/10.1093/ehjqcco/qcad040

In the originally published version of this manuscript, the author Anzhela Soloveva was inadvertently omitted from the online version. The PDF and printed versions included the correct list of names. The incorrect ORCID was also given for this author. The correct ORCID is 0000–0002-0013–0660.

In addition to this, the author contributions have been expanded to say that ‘A.S. produced Figure 2.’

These errors have been corrected online.

