# Supplementary material for: Cohort profile: Evaluation of the Methods and Management of Acute Coronary Events (EMMACE) longitudinal cohort
Source: Eur Heart J Qual Care Clin Outcomes. 2023 Jul 14;9(5):442–6. doi: 10.1093/ehjqcco/qcad040 (PMC10405135; doi:10.1093/ehjqcco/qcad040)
Supplement: qcad040_Supplemental_File [file qcad040_supplemental_file.docx]

Table S1: Data proforma showing variables in the EMMACE dataset and data sources

| **Variable** | **Source of data** | **Variable** | **Source of data** |
| --- | --- | --- | --- |
| In patient hospitalisations | HES | **Comorbidities (Diagnostic criteria (ICD codes)** | MINAP, HES |
| Age at admission | HES | Hypertension | MINAP, HES |
| Sex | HES | Atrial Fibrillation | MINAP, HES |
| Body Mass Index (BMI) | HES | Stroke | MINAP, HES |
| Ethnicity | HES | Angina | MINAP, HES |
| Index of multiple deprivation (IMD) | HES | Peripheral vascular disease | MINAP, HES |
| Smoking status | HES | Valvular heart disease | MINAP, HES |
| Employment status | HES | Congestive heart failure | MINAP, HES |
| Admission diagnoses | HES | Diabetes mellitus | MINAP, HES |
| Operations | HES | Renal failure | MINAP, HES |
| Length of stay | HES | Asthma or COPD | MINAP, HES |
| Care provider | HES | Heart failure | MINAP, HES |
| Revascularisation procedures | MINAP, HES | Cancer | HES |
| **Discharge medication** |  | Hospital provider | HES |
| Beta blockers | MINAP | Diagnoses (STEMI/ NSTEMI, Unstable Angina) | MINAP |
| Angiotensin-converting enzyme inhibitors | MINAP | **Patient reported outcome measures** |  |
| Statins | MINAP | EQ-5D-3L | EMMACE-3, -4, -XL, XXL |
| Aspirin | MINAP | EQ-VAS | EMMACE-3, -4 , -XL, XXL |
| Clopidogrel | MINAP | Beliefs about medicines questionnaire | EMMACE-3 & -4 |
| Angiotensin II receptor blockers | MINAP | PRISMA-7 frailty measure | EMMACE-XXL |
| Ticagrelor | MINAP | Satisfaction with Information about Medicines Scale | EMMACE-3 & -4 |
| Prasugrel | MINAP | Brief Illness perception | EMMACE-3 & -4 |
| Killip class | MINAP | Care Quality Commission Pickering Inpatient questionnaire | EMMACE-3 & -4 |
| **Treatments** |  | Single Question Medicine Adherence | EMMACE-3 & -4 |
| Percutaneous coronary Intervention (PCI) | MINAP, HES | Medications | EMMACE -3,4, EMMACE XL, XXL |
| Coronary artery bypass graft (CABG) |  | Mortality | ONS |
| Thrombolysis | MINAP |  |  |
| Pharmacoinvasive strategy | MINAP |  |  |
| Cardiac rehabilitation | MINAP |  |  |
| **Physical activity** | EMMACE-3, -4 |  |  |

COPD, chronic obstructive pulmonary disease; NSTEMI, non-ST segment elevation myocardial infarction; STEMI, ST segment elevation myocardial infarction; PROMs, patient-reported outcome measures; MINAP, the Myocardial Ischemia National Audit Project; EMMACE, the Evaluation of the Methods and Management of Acute Coronary Events; EQ-5D-3L, the 3-level EuroQol 5-dimension; EQ-VAS, EQ visual analogue scale; HES, Hospital Episodes Statistics; ONS, Office for National Statistics.
